# Polymorphisms of TP53 codon 72 with breast carcinoma risk: evidence from 12226 cases and 10782 controls

**DOI:** 10.1186/1756-9966-28-115

**Published:** 2009-08-14

**Authors:** Wenlei Zhuo, Yunsong Zhang, Zhaolan Xiang, Lei Cai, Zhengtang Chen

**Affiliations:** 1 Institute of Cancer, Xinqiao Hospital, Third Military Medical University, Chongqing, PR China; 2 Department of Thoracic Surgery, the 254thHospital, Tianjin, PR China; 3 Department of Otolaryngology, Southwest Hospital, Third Military Medical University, Chongqing, PR China; 4 Institute of Hepatobiliary Surgery, Southwest Hospital, Third Military Medical University, Chongqing, PR China

## Abstract

**Background:**

Previously, TP53 codon 72 polymorphisms have been implicated as risk factors for various cancers. A number of studies have conducted on the association of TP53 codon 72 polymorphisms with susceptibility to breast carcinoma and have yielded inconclusive results. The aim of the present study was to derive a more precise estimation of the relationship.

**Methods:**

We conducted a search in the Medline, EMBASE, OVID, Sciencedirect, and Chinese National Knowledge Infrastructure (CNKI) without a language limitation, covering all papers published up to Jan 2009. The associated literature was acquired through deliberate searching and selected based on the established inclusion criteria for publications.

**Results:**

A total of seventeen case-control studies, including 12226 cases and 10782 controls, met the included criteria and thus were selected. Ultimately, the relevant data were extracted and further analyzed using systematic meta-analyses. Overall, no associations of TP53 codon 72 polymorphisms with breast carcinoma were observed (for Arg/Arg vs Pro/Pro: OR = 1.20; 95%CI = 0.96–1.50; for dominant model: OR = 1.12; 95%CI = 0.96–1.32; for recessive model: OR = 1.13; 95%CI = 0.98–1.31). In the subgroup analysis by ethnicity, statistically similar results were obtained when the data were stratified as Asians, Caucasians and Africans.

**Conclusion:**

Collectively, the results of the present study suggest that *TP53 codon 72 *polymorphisms might not be a low-penetrant risk factor for developing breast carcinoma.

## Backgrounds

Breast cancer is the second leading cause of cancer death in women, exceeded only by lung cancer in the world [1]. It is believed that some epidemic factors such as Oral contraceptive use [2]; obesity [3] and hyperinsulinemia [4] are probable factors increasing risks of developing breast carcinoma. Although many individuals exposed to these risk factors, breast cancer develops only in a small group of exposed people, implying that genetic factors might contribute to the carcinogenic mechanisms and complex interactions between many genetic and environmental factors might be the major cause of breast cancer.

Previously, a number of studies indicate that family history is a risk factor for breast cancer [5], indicating the possible roles for genetic variations on the increased susceptibility to breast cancer. Recent published meta-analyses suggest that polymorphisms of Fok1 [6], XRCC1 codon 399[7] and methylenetetrahydrofolate reductase[8] might have a significant association with increased breast cancer risk. Nevertheless, conversely, some meta-analysis failed to suggest a marked association of increased susceptibility to breast cancer with polymorphisms of some genes, such as Estrogen receptor alpha [9], CYP1A1 [10] and base-excision repair pathway genes [11].

Recently, a growing body of research has conducted on the association of breast cancer risk with tumour suppressors. TP53, one of the most extensive studied genes as a tumor suppressor, has been thought to have a critical function in cell cycle regulation. In case of its mutation, this regulation could be lost, resulting in cell proliferation without control and development of cancer. Previously, TP53 mutation has been indicated to associate with risks of a number of cancers such as lung cancer[12], breast cancer [13] and colorectal cancer [14]. The loss of TP53 gene could damage its DNA-binding properties and transcription factor function, thus leading to aberrant cell proliferation. In human populations, the TP53 gene is polymorphic at amino acid 72 of the protein that it encodes.

Recently, much attention has been focused on possible associations of TP53 polymorphisms and cancer risks. The most informative polymorphism in TP53 gene is located in exon 4 at codon 72, which encodes two distinct functional allelic forms arginine (Arg) and proline (Pro) because of a transversion G to C [15], resulting in different biochemical and biological protein features. Consequently, three distinct genotypes were created, namely, homozygous for arginine (Arg/Arg), homozygous for proline (Pro/Pro), and heterozygous (Arg/Pro). Previously, Arg variant has been thought to increase susceptibility to gastric cancer[16] and Arg homozygosity might contribute to cervical cancer [17]. Nevertheless, Pro homozygosity might have an association with lung [18] and hepotocellular cancer [19] risk. The heterozygous genotype Arg/Pro has been implicated as a risk factor for bladder cancer [20].

In recent literature, inconclusive data regarding TP53 codon 72 were found in some cancers, such as gastric cancer in which controversial conclusions were obtained in Asians [21] and in individuals from Northern Brazil [22]. Similarly, up to date, published data on the possible association of TP53 codon 72 polymorphism with breast carcinoma have also generated controversial and inconclusive results. To the best of our knowledge, whether TP53 codon 72 polymorphism could increase breast cancer risk remains largely uncertain. To clarify this association may help us better understand the possible risk of breast cancer and therefore contribute to its prevention.

As a single study may have been underpowered in clarifying the relationship of TP53 codon 72 polymorphisms with breast carcinoma susceptibility, in the present study we performed evidence-based quantitative meta-analyses that can increase statistical power to address the association.

## Materials and methods

### Literature search strategy for identification of the studies

We carried out a search in the Medline, EMBASE, OVID, Sciencedirect, and Chinese National Knowledge Infrastructure (CNKI) without a language limitation, covering all papers published up to Jan 2009, with a combination of the following keywords: *TP53, P53, codon 72, breast, carcinoma, neoplasm, tumor, cancer *and *polymorphism*. The keywords were paired each time in order to get more relevant information. For example, the word "breast" was always kept and others were substituted in different moments.

We evaluated potentially associated publications by checking their titles and abstracts and then procured the most relevant publications for a closer examination. Moreover, the reference lists of the selected papers were also screened for other potential articles that possibly have been missed in the initial search. The following criteria were used for the literature selection for the further meta-analysis:

1. Studies concerning the association of TP53 codon 72 polymorphism with breast carcinoma;

2. Case–control or cohort studies;

3. Papers presenting the breast cancer diagnoses and the sources of cases and controls;

4. Articles offering the size of the sample, odds ratios (ORs) and their 95% confidence intervals (CIs) or the information that can help infer the results;

5. The number of individuals homozygous for arginine (Arg/Arg), proline (Pro/Pro) and heterozygous (Pro/Arg) in breast cancer cases and controls should be offered;

6. The methods of data collection and analysis should be statistically acceptable.

Accordingly, the following exclusion criteria were also used:

1. The design and the definition of the experiments were obviously different from those of the selected papers.

2. The source of cases and controls and other essential information were not offered;

3. The genetic distribution of the control group was inconsistent with Hardy-Weinberg equilibrium (HWE).

4. Reviews and duplicated publications.

After searching, we reviewed all papers in accordance with the criteria defined above for further analysis.

### Data extraction

Data were carefully extracted from all eligible publications independently by two of the authors according to the inclusion criteria mentioned above. For conflicting evaluations, an agreement was reached following a discussion. If a consensus could not be reached, another author was consulted to resolve the dispute and then a final decision was made by the majority of the votes. The extracted information was entered into a database. For data not provided in the main text, the relevant information was obtained by contacting corresponding authors as possible as we could.

### Statistical analysis

The odds ratio (OR) of TP53 codon 72 polymorphisms and breast cancer risk was estimated for each study. The pooled ORs were performed for additive model (Arg/Arg vs Pro/Pro), dominant model (Arg/Arg+Arg/Pro versus Pro/Pro) and recessive model (Arg/Arg versus Arg/Pro+Pro/Pro), respectively. For detection of any possible sample size biases, the OR and its 95% confidence interval (CI) to each study was plotted against the number of participants respectively. A Chi-square based Q statistic test was performed to assess heterogeneity. If the result of the heterogeneity test was *P *> 0.05, ORs were pooled according to the fixed-effect model (Mantel-Haenszel), Otherwise, the random-effect model (DerSimonian and laird) was used. The significance of the pooled ORs was determined by Z-test. The HWE was assessed via Fisher's exact test.

Publication bias was assessed by visual inspection of funnel plots[23], in which the standard error of log (OR) of each study was plotted against its log (OR). An asymmetric plot indicates a possible publication bias. The symmetry of the funnel plot was further evaluated by Egger's linear regression test[24]. In addition, fail-safe number for *P *= 0.05 (Nfs_0.05_) [25] for the evaluation of the reliability of meta-analysis, defined as the number of negative results that could reverse the significant findings, was also used to estimate the robustness of the meta analysis. Statistical analysis was undertaken using the program Review Manager 4.2 and SAS 8.1 software.

## Results

### Study characteristics

**Figure 1:**
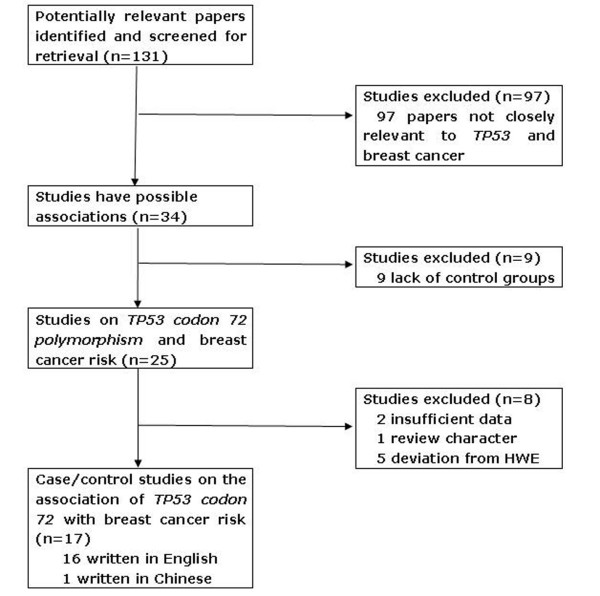
**The flow diagram of included/excluded studies**.

A total of 131 studies regarding TP53 codon 72 with respect to breast cancer were searched and screened for retrieval, of which 97 irrelevant studies were excluded. Then, 9 studies [26-34] were excluded because each of them did not contain a control group. Next, of the remaining 25 studies, 2 studies[35,36] were excluded due to their insufficient data and 1 study [37] owing to its review characteristic. Afterwards, another 5 studies [38-42] were excluded because the genetic distributions of the control groups were not in agreement with HWE. Lastly, 17 case-control studies were selected (Figure 1). Of the all included 17 studies, 16 were written in English [43-58] and 1 [59] in Chinese.

We established a database according to the extracted information from each article. The relevant information was listed in Table 1. According to the lists, the first author and the number and characteristics of cases and controls for each study as well as other necessary information were presented. As shown in Table 2, the distributions of TP53 codon 72 genotype of the included studies were also presented. The controls of the included studies were all in accordance with HWE.

**Table 1:** Characteristics of studies included in the meta-analysis

First Author	Publication Year	Number of Cases	Number of Controls	Types of Cases	Type of controls	Method	Country	Ref. No.
Själander	1996	212	689	Ductal carcinoma (>80% of the total cases)	Pooled individuals from several controls	PCR-RFLP	Sweden	43
Weston	1997	81	147	NS	NS	AS-PCR	USA	44
Li	2002	28	50	NS	50 healthy people (age-matched)	AS-PCR	China	45
Wang-Gohrke	2002	577	579	NS	NS	PCR-RFLP	Germany	46
Buyru	2003	115	76	NS	76 healthy people	PCR-RFLP	Turkey	47
Huang	2003	200	282	NS	282 healthy people	PCR-CTPP	China	48
Katiyar	2003	77	41	77 Sporadic breast cancer	41 Normal healthy women (age-matched)	PCR-RFLP	India	49
Mabrouk	2003	30	49	NS	NS	PCR-RFLP	Tunisia	50
Kalemi	2005	42	51	NS	51 healthy women	PCR-RFLP	UK	51
Tommiska	2005	1827	736	888 unselected breast cancer; 939 familial breast cancer	736 healthy population	TaqMan	Sweden	52
Baynes	2007	2023	2197	NS	NS	TaqMan	UK	53
Gochhait	2007	576	243	243 sporadic breast cancer; 333 unrelated breast cancer	243 healthy females (ethnically and geographically matched)	PCR-RFLP	India	54
Khadang	2007	221	205	221 sporadic breast cancer	205 healthy blood donors	AS-PCR	Iran	55
Schmidt	2007	5191	3834	NS	NS	TaqMan+ PCR-RFLP	UK	56
Sprague	2007	1912	1527	1708 invasive breast cancer; 204 in situ breast cancer	1527 drivers (< 65 year) and roster of Medicare beneficiaries (65–74 year)	TaqMan	USA	57
Zhang	2007	84	168	NS	168 healthy controls (geographic- and age-matched)	PCR-RFLP	China	59
Akkiprik	2008	95	107	NS	107 age-matched healthy controls	PCR-RFLP	Turkey	58

AS-PCR: Allele Specific PCR; RFLP: restriction fragment length polymorphism; CTPP: confronting two-pair primers; NS: not specified

**Table 2:** Distribution of TP53 codon 72 genotype among breast cancer cases and controls included in the meta-analysis

First author	Cases			Controls			HWE (control)	
	
	Arg/Arg	Arg/Pro	Pro/Pro	Arg/Arg	Arg/Pro	Pro/Pro	Chi-square	*P*
Själander	24	93	95	61	253	375	3.681	0.055
Weston (Caucasian)	6	27	32	3	42	72	1.189	0.276
Weston (African)	6	9	1	12	14	4	0.001	0.979
Li	11	11	6	10	26	14	0.109	0.741
Wang-Gohrke	282	221	49	300	203	40	0.485	0.486
Buyru	64	39	12	21	43	12	1.657	0.198
Huang	64	100	36	114	138	30	1.545	0.214
Katiyar	20	51	6	9	24	8	1.205	0.272
Mabrouk	18	9	3	19	26	4	1.432	0.231
Kalemi	26	13	3	10	32	9	3.326	0.068
Tommiska	825	617	109	403	278	52	0.183	0.669
Baynes	1107	768	148	1177	854	166	0.414	0.520
Gochhait	86	109	48	76	160	97	0.413	0.521
Khadang	83	109	29	75	90	40	1.873	0.171
Schmidt	2797	2008	386	2024	1523	287	0.001	0.983
Sprague	823	570	89	705	490	83	0.03	0.862
Zhang	21	45	17	47	87	33	0.406	0.524
Akkiprik	25	50	20	46	49	12	0.038	0.846

### Test of heterogeneity

We analyzed the heterogeneity of Arg/Arg versus Pro/Pro and dominant model (Arg/Arg+Arg/Pro versus Pro/Pro) as well as recessive model (Arg/Arg versus Arg/Pro+Pro/Pro). As shown in Table 3, the heterogeneity for the overall data was significant in each of the above three models respectively because the P values were less than 0.05 for Q-tests. Thus, random-effect models were utilized for the meta-analyses.

**Table 3:** Main results of the pooled data in the meta-analysis

	No. of cases/controls	Arg/Arg vs Pro/Pro			(Arg/Arg+Arg/Pro) vs Pro/Pro			Arg/Arg vs (Arg/Pro+Pro/Pro)		
		
		OR (95%CI)	P	P (Q-test)	OR (95%CI)	P	P (Q-test)	OR (95%CI)	P	P (Q-test)
Random-effect model										
Total	12226/10782	1.20 (0.96–1.50)	0.11	0.000	1.12 (0.96–1.32)	0.14	0.01	1.13 (0.98–1.31)	0.10	0.000
Caucasian	11549/9830	1.15 (0.91–1.44)	0.24	0.001	1.11 (0.95–1.30)	0.17	0.06	1.09 (0.93–1.27)	0.28	0.000
Asian	631/873	1.36 (0.61–3.03)	0.45	0.000	1.19 (0.67–2.10)	0.55	0.006	1.22 (0.72–2.05)	0.46	0.002
African	46/79	1.46 (0.38–5.62)	0.58	0.76	1.12 (0.31–4.10)	0.86	0.45	1.60 (0.63–4.06)	0.32	0.22
Fixed-effect model										
Total	12226/10782	1.09 (0.99–1.20)	0.10	0.000	1.09 (0.99–1.19)	0.06	0.01	1.04 (0.99–1.10)	0.13	0.000
Caucasian	11549/9830	1.07 (0.96–1.18)	0.24	0.001	1.08 (0.98–1.19)	0.12	0.06	1.03 (0.98–1.09)	0.25	0.000
Asian	631/873	1.27 (0.94–1.71)	0.12	0.000	1.16 (0.89–1.51)	0.26	0.006	1.15 (0.92–1.44)	0.22	0.002
African	46/79	1.47 (0.39–5.62)	0.57	0.76	1.17 (0.33–4.14)	0.80	0.45	1.67 (0.80–3.48)	0.17	0.22

### Meta-analysis results

Table 3 lists the main results of the meta-analysis. No evidence showed that individuals who carry Arg allele have an increased or decreased risk of breast carcinoma compared with those who carry Pro allele.

In the present study, a total of 17 studies were included. Nevertheless, the study conducted by Weston et al. [44] concerned both Caucasians and Africans. Thus, the data were extracted respectively and further assessed by Revman 4.2 software. Consequently, the following results reported 18 studies.

As shown in Table 3, for Arg/Arg vs Pro/Pro, the data available for our meta-analysis were obtained from 18 case-control studies of 7377 cases and 6450 controls, of which 6288 cases and 5112 controls had the Arg/Arg genotype and 1089 cases and 1338 controls had the Pro/Pro genotype of the TP53 codon 72. The overall OR was 1.20 (95% CI = 0.96–1.50) and the test for overall effect Z value was 1.58 (*P *> 0.05). For dominant model (Arg/Arg+Arg/Pro versus Pro/Pro), the data available for our meta-analysis were obtained from 18 case-control studies containing 12226 cases and 10782 controls, of which 11137 cases and 9444 controls had the combined genotypes of Arg/Arg and Arg/Pro, while 1089 cases and 1338 controls had the homozygote Pro/Pro genotype. The overall OR was 1.12 (95% CI = 0.96–1.32) and the test for overall effect Z value was 1.47 (*P *> 0.05). Similarly, for recessive model (Arg/Arg versus Arg/Pro+Pro/Pro), the data were extracted from the 18 case-control studies concerning 12226 cases and 10782 controls, of which 6288 cases and 5112 controls had the wild-type homozygote Arg/Arg genotype while 5938 cases and 5670 controls had the combined variant genotypes (Arg/Pro and Pro/Pro) of the TP53 codon 72. The overall OR was 1.13 (95% CI = 0.98–1.31) and the test for overall effect Z value was 1.65 (*P *> 0.05).

Considering the possible impact of ethnic variation on the results, we conducted subgroup analysis concerning Asians, Caucasians and Africans, respectively. Likewise, the subgroup analyses failed to suggest marked association between TP53 codon 72 polymorphisms and breast cancer risk in Asians, Caucasians and Africans.

### Sensitivity analysis

In order to compare the difference and evaluate the sensitivity of the meta-analyses, we also presented the results of the fixed-effect models as listed in Table 3. In all, the results were not significantly different between the two models, suggesting the robustness of the meta-analyses. Moreover, we also conducted one-way sensitivity analysis[60] to evaluate the stability of the meta-analysis. The statistical significance of the results was not altered when any single study was omitted (data not shown), confirming the stability of the results. Hence, results of the sensitivity analysis suggest that the data in this meta-analysis are relatively stable and credible.

### Bias diagnostics

Funnel plots were created for assessment of possible publication biases. Then, Egger's linear regression tests were used to assess the symmetric of the plots. As shown in Table 4, for the dominant model, the data suggest that the funnel plot is symmetrical. However, for the additive and recessive model, the results indicate possible asymmetric of the funnel plots. Therefore, we further calculated the Nfs_0.05 _for evaluation of the stability of the results. Consequently, the Nfs_0.05 _were 237, 143 and 271 for additive, dominant model and recessive model respectively, which were more than five times of the number of the included studies, suggesting that the results of these meta-analyses are relatively stable and the publication biases might not have an evident influence on the results of the meta-analyses.

**Table 4:** Publication bias tests (Egger's linear regression test and Nfs_0.05_) for TP53 codon 72 polymorphisms

Genetic type	Coefficient	Standard Error	t	P value	95% CI of intercept	Nfs_0.05_
Arg/Arg vs Pro/Pro	2.757	1.0434	2.641	0.018	(0.544, 4.970)	237
Arg/Arg+Arg/Pro vs Pro/Pro	1.172	0.659	1.778	0.094	(-0.225, 2.570)	143
Arg/Arg vs Arg/Pro+Pro/Pro	2.726	1.183	2.305	0.034	(0.219, 5.234)	271

## Discussion

In the present study, the results of meta-analyses showed that individuals with TP53 codon 72 polymorphism might not have significant associations with increased or decreased susceptibility to breast carcinoma.

A previous meta-analysis conducted by Koushik et al. [61] regarding cervical cancer suggests that homozygote Arg/Arg genotype increases susceptibility to both squamous cell carcinoma and adenocarcinoma. While another meta-analysis [62] indicates that Arg/Arg genotype only associates with increased risk of cervical adenocarcinoma but not squamous cell carcinoma. Then, Sousa et al. [63] failed to demonstrate Arg/Arg genotype as a risk marker for the development of cervical lesions in most of European countries. Conversely, nonassociations of TP53 codon 72 polymorphism with lung carcinoma [64] and gastric cancer [65] risk were found by meta-analysis. Nevertheless, An updated meta-analysis concerning lung cancer implied that Pro allele is a low-penetrant risk factor for developing lung cancer [66]. Thus, whether TP53 codon 72 polymorphism contributes to susceptibility to cancers varies in different types of cancer. In the present study, no evidence showed TP53 codon 72 polymorphism as a risk factor for breast cancer.

The underlying mechanisms by which TP53 polymorphism influences cancer risk are not fully understood. TP53 is the most frequently investigated gene that is often mutated in a variety of cancers. Nevertheless, several single-nucleotide polymorphisms have been studied and reported in TP53 gene [67]. The polymorphism of TP53 codon 72 occurs in a proline-rich region that is thought to play a critical role in the growth suppression and apoptotic functions of TP53 protein [68]. The two polymorphic variants differ in their capability of binding the transcriptional protein, activating transcription and suppressing the transformation of some primary cells [69]. For example, Arg variant might induce cell apoptosis and suppress transformation more efficiently than Pro variant do, which may be due to the ability of the Arg variant to localize in mitochondria that regulates the release of cytochrome c into cytosol. However, the present meta-analysis indicates that neither Arg nor Pro carriers may have a significant association with breast cancer risk. It is likely that TP53 codon 72 polymorphisms rarely affect the tumorigenesis and progression of breast carcinoma. Considering that the same polymorphism may play different roles in cancer susceptibility among different ethnic populations and the frequencies of single nucleotide polymorphisms may be different ethnicity, we stratified the data by race into three groups concerning Asians, Caucasians or Africans, respectively. Ultimately, statistically similar results were obtained, confirming nonassociation of TP53 codon 72 polymorphism with breast cancer risk.

A well-known risk factor, HPV infection, is thought to have an association with increased susceptibility to some cancers such as cervical [70] and oral cancer [71]. Evidence suggests that P53Arg72 protein may be more susceptible than P53Pro72 protein to HPV mediated degradation, thus increasing risk of HPV associated cancers [17]. Growing body of literature indicates HPV infection as a possible risk factor for breast cancer [72]. However, we did not further investigate the possible association of HPV infection with TP53 codon 72 polymorphism due to the insufficient data in the primary included studies.

Heterogeneity is a potential problem when interpreting the results of meta-analysis [73]. In the present study, significant between-study heterogeneity existed in overall comparisons. Nevertheless, when the data were stratified by race, the heterogeneity was decreased or removed, suggesting that differences of genetic backgrounds and the environment existed among different ethnicities. In the present meta-analysis, we excluded the studies in which the control groups were deviate from HWE. Thus, the between-study heterogeneity might be reduced. Moreover, random-effect models were used for combination of the data. Accordingly, the results may be credible and stable although the heterogeneity seemed evident.

Some limitations might be included in this study. First, in this meta-analysis, most published studies and papers written in English or Chinese were searched. Moreover, although papers written in some other languages, cited by PubMed, were also searched, it is possible that some related published or unpublished studies that might meet the inclusion criteria were missed. Hence, some inevitable publication biases might exist in the results, though the Nfs_0.05 _showed no remarkable publication biases in the meta-analyses. Second, in the subgroup analysis, the number of studies regarding Africans was relatively limited. It may be underpowered to explore the real association. Thus, the results may be interpreted with caution. Third, whether the experimental and control groups were from the same socio-economic status or the same geographic area have not been clearly presented in some of the included original papers, leading to any possible biases. Furthermore, the sample sizes of some included studies are rather small, which might be one of the reasons contributing to the between-study heterogeneity. Therefore, a number of further studies with large sample sizes with well-matched controls are required. Besides, gene-gene and gene-environment interactions should also be considered in the further studies.

In summary, despite the limitations, the results of the present meta-analysis suggest that genetic variations of TP53 codon 72 may not have a marked association with breast cancer risk.

## Competing interests

The authors declare that they have no competing interests.

## Authors' contributions

WZ and YZ conceived of the study, and carried out the analysis of the literatures and drafted the manuscript. ZX carried out the collection of the literatures. LC helped with the statistical analysis and manuscript drafting. ZC and WZ conceived of the study, and participated in its design and coordination and helped to draft the manuscript. All authors read and approved the final manuscript.
